# The interrelationship between food security, climate change, and gender-based violence: A scoping review with system dynamics modeling

**DOI:** 10.1371/journal.pgph.0000300

**Published:** 2023-02-24

**Authors:** Pooja Agrawal, Lori Ann Post, Janis Glover, Denise Hersey, Piya Oberoi, Brian Biroscak

**Affiliations:** 1 Department of Emergency Medicine, Yale University School of Medicine, New Haven, Connecticut, United States of America; 2 Buehler Center for Health Policy and Economics, Northwestern University Feinberg School of Medicine, Chicago, Illinois, United States of America; 3 Cushing/Whitney Medical Library, Yale University, New Haven, Connecticut, United States of America; 4 Dana Medical Library, University of Vermont, Burlington, Vermont, United States of America; 5 Piya Oberoi, Wesleyan University, Middletown, Connecticut, United States of America; New York University Grossman School of Medicine, UNITED STATES

## Abstract

Gender-based violence (GBV) is a global public health and human rights problem that is exacerbated by social and environmental stressors for a multitude of interpersonal, cultural, and economic reasons. Through sudden disruptions in the microclimate of a region, climate shocks often have a negative impact on food security, which correlates with increases in GBV. Associations between the various combinations of GBV, climate change, and food insecurity have been documented in the growing international literature, but questions remain about these associations that require further clarification. The impact of the COVID-19 pandemic caused by SARS-CoV-2 provides insight through a real time demonstration into these interactions. This review of the global literature examines the interplay between GBV, climate change, and food insecurity—including recent literature regarding the COVID-19 pandemic. This review covers original research studies employing both quantitative and qualitative methodology, those that conducted secondary analyses of existing data sources and perspective pieces derived from observed evidence. An additional analytic layer of system dynamics modeling allowed for the integration of findings from the scoping review and discovery of additional insights into the interplay between disasters, food insecurity, and GBV. Findings from this review suggest that the development and adaptation of evidence-based, focused interventions and policies to reduce the effects of climate shocks and bolster food security may ultimately decrease GBV prevalence and impact.

## Introduction

Climate shocks, sudden disruptions to the extremes of temperature or precipitation in the microclimate of a region, are becoming more common around the world [1,2]. Examples of climate shocks include sudden periods of drought, flooding, and extreme heat, often occurring with little warning, which precludes the ability to avoid them. Developing countries with diminished reserves, a limited ability to adapt, and vulnerable populations are often disproportionately affected by the stress of extreme climate events on the population [3]. These climate shocks are seen as a direct result of climate change and are likely to continue increasing in frequency worldwide [4].

Food security, as defined by the United Nations is “when all people, at all times have physical and economic access to sufficient, safe, and nutritious food to meet their dietary needs and food preferences for an active and healthy life” [5]. Much of the world suffers from food insecurity, which leads to increased poverty and negatively impacts agricultural systems and social structures [6–8]. Climate shocks create a strain on food production, transportation infrastructure, and access to food for much of a vulnerable population [9]. People become less able to feed their families and make their livelihoods in the manner with which they did prior to the onset of the climate shock. In parts of the world with little cushion to absorb the personal as well as socio-economic impacts of food insecurity regardless of cause, small but dramatic changes in the climate of a region can have significant negative effects on societal dynamics [10]. Food insecurity is concentrated in sub-Saharan Africa and South Asia, where up to 30% of the population is undernourished [11] and where the population has the smallest reserve to deal with diminished access to food.

Women and female children are often the first to be neglected and negatively impacted during periods of food insecurity [12]. There are many economic, cultural, political, and structural explanations for this. Women are often less able to generate independent income, leading to increased reliance on male household members and exacerbated gender inequality [13]. Scarce resources are typically channeled to men and bread-winning males of a family in order to allow them to continue working [14]. When men are psychologically and socially stressed due to insufficient food stemming from climate shocks and subsequent food insecurity, they often take out their frustrations on women [15]. Gender-based violence (GBV) includes a variety of injurious behaviors that are directed at women and girls simply on the basis of their gender. GBV takes many forms, including but not limited to psychological abuse, physical violence, sexual violence, coercion, survival sex, female genital mutilation, selective malnourishment or undernourishment of female children, and femicide. GBV is often a manifestation of stressors and frustrations caused by food insecurity. As food insecurity increases, so do gender inequalities and violence against women [13,16,17].

The COVID-19 pandemic caused by SARS-CoV-2 has revealed challenges that demonstrate the impact of climate change on many aspects of life, such as the greater emergence of infectious diseases, the substantial disruption to the global food supply chain, and disproportionate impact on communities of color, to name a few [18–21]. The parallels between the pandemic and the anticipated effects of ongoing climate change, including the level of complexity of each challenge, the prolonged lag time until situational improvement, the irreversibility of the impact, the differential effect on vulnerable subpopulations, and the splintering of global alliances caused by limited resources and a refocus on nationalism despite the need for collective action, all provide an early warning of future potential evolving disasters [20,22]. COVID-19 has disrupted supply chains of all forms, including those used for food distribution. This further exacerbates food insecurity across the globe and affects food production, availability, access, utilization, and stability [23,24]. Stay-at-home orders add to this food insecurity challenge, as well as to the prevalence of GBV [25]. The heightened stress and uncertainty stemming from the potential for future economic insecurity represented by food insecurity can increase the incidence and frequency of GBV [26,27]. The COVID-19 pandemic, like other disasters and times of economic and personal stress, has done this [27].

Bivariate associations between climate shocks and food insecurity, as well as food insecurity and gender-based violence have been explored. For example, a cross-country analysis demonstrated that smaller households experiencing a climate shock were more likely to be food insecure, and large-scale survey analyses examined the hypothesis that food insecurity impacts sexual violence through risky sexual behavior [28,29]. However, the inter-relatedness of all three of these concepts has not been fully investigated [16,30]. The COVID-19 pandemic has given us a real-time perspective on how outbreaks, a natural extension of climate change, can exacerbate the elements described here. With the stated evidence that both climate shocks and food insecurity are increasing worldwide, it naturally follows that GBV will also likely increase. Given the immense psychosocial and socio-economic impact of violence against women, there needs to be further investigation into ways to decrease GBV as a direct result of food insecurity caused by climate shocks and disasters in general—including pandemics.

### Objectives

The primary objective of this scoping review was to examine the extent, range, and nature of scholarly activity regarding the interrelationship between food security, climate change, and gender-based violence, inclusive of literature regarding pandemics. Our secondary objective was to summarize and disseminate scholarly findings to policy makers, funders, and researchers; and our tertiary objective was to identify research gaps in the existing literature. Our overall goal: to encourage concerted action by documenting and sharing the ‘mental models’ of scholars working in this field, was achieved through a strategic combination of evidence synthesis, content analysis, and system dynamics modeling.

## Methods

We performed a scoping review in accordance with the methodology for Joanna Briggs Institute scoping reviews [31], which draws upon two other influential scoping review frameworks [32]. A ‘scoping review’ has been defined as an attempt to map:

… the key concepts underpinning a research area and the main sources and types of evidence available, and can be undertaken as stand-alone projects in their own right, especially when an area is complex or has not been reviewed comprehensively before [33,34].

Because of their broad nature, scoping reviews are particularly valuable for assembling evidence from disparate or heterogeneous sources (e.g., peer-reviewed journal articles, government reports, technical guides, etc.) [31]. They are better suited than systematic reviews to initially identify types of available evidence and examine how research is conducted on a certain topic or field [35]. Scoping review methods are typically reported under the following rubrics: inclusion criteria, types of sources, search strategy, and extraction of results.

### Inclusion criteria

We sought to understand what types and range of evidence have been reported regarding the interrelationship between food security, climate change, and gender-based violence. Sources were included in this scoping review if they addressed this question, our aforementioned objectives, and the PCC mnemonic: Population, Concepts, Context [31].

#### Population

Our population of interest consisted of victims of gendered and sexualized violence (regardless of age). We did not specify any other exclusion criteria regarding important characteristics of study participants (e.g., nature of relationship between GBV victim and perpetrator, co-morbidities, etc.).

#### Concepts

The three core concepts examined by our initial scoping review were food security, climate change, and gender-based violence. The definition of *food security* used in USAID’s “Food Security Country Framework” focuses on three distinct but interrelated elements: food availability, food access, and food utilization/consumption [36]. *Climate change* referred to any significant change in the measures of climate (e.g., temperature, precipitation, wind effects, etc.) lasting for an extended period of time, or its presumed effects (e.g., flooding, drought, etc.) [37]. *Gender-based violence* was defined as violence (e.g., physical acts, threats of acts, coercion, etc.) directed against a woman because she is a woman or that affects women disproportionately [38]. While there was variability in terminology, we used broad definitions of each concept in order to maintain inclusivity.

#### Context

We did not specify any inclusion criteria regarding the context such as cultural factors, geographic location, etc., as this was not germane to our research question. However, sources had to be published in the English language and the types of evidence could not include lay publications (e.g., newspapers, magazines, etc.).

### Types of sources

The following databases were searched for relevant studies: MEDLINE (Ovid), Embase (Ovid), Global Health (Ovid), BIOSIS, Web of Science (Thomson Reuters), CAB Direct, Africa Wide, PAIS. Two selected grey literature sources—IRIS (Institutional Repository for Information Sharing, World Health Organization) and NGO custom search in Google—were also explored. The search strategy was not limited by study design.

### Search strategy

The databases were searched using both controlled vocabulary words and synonymous free text words to capture the concepts of gender disparities, food security, and climate change (S1 File). The search strategies were adjusted for the syntax appropriate for each database/platform.

**Fig 1:**
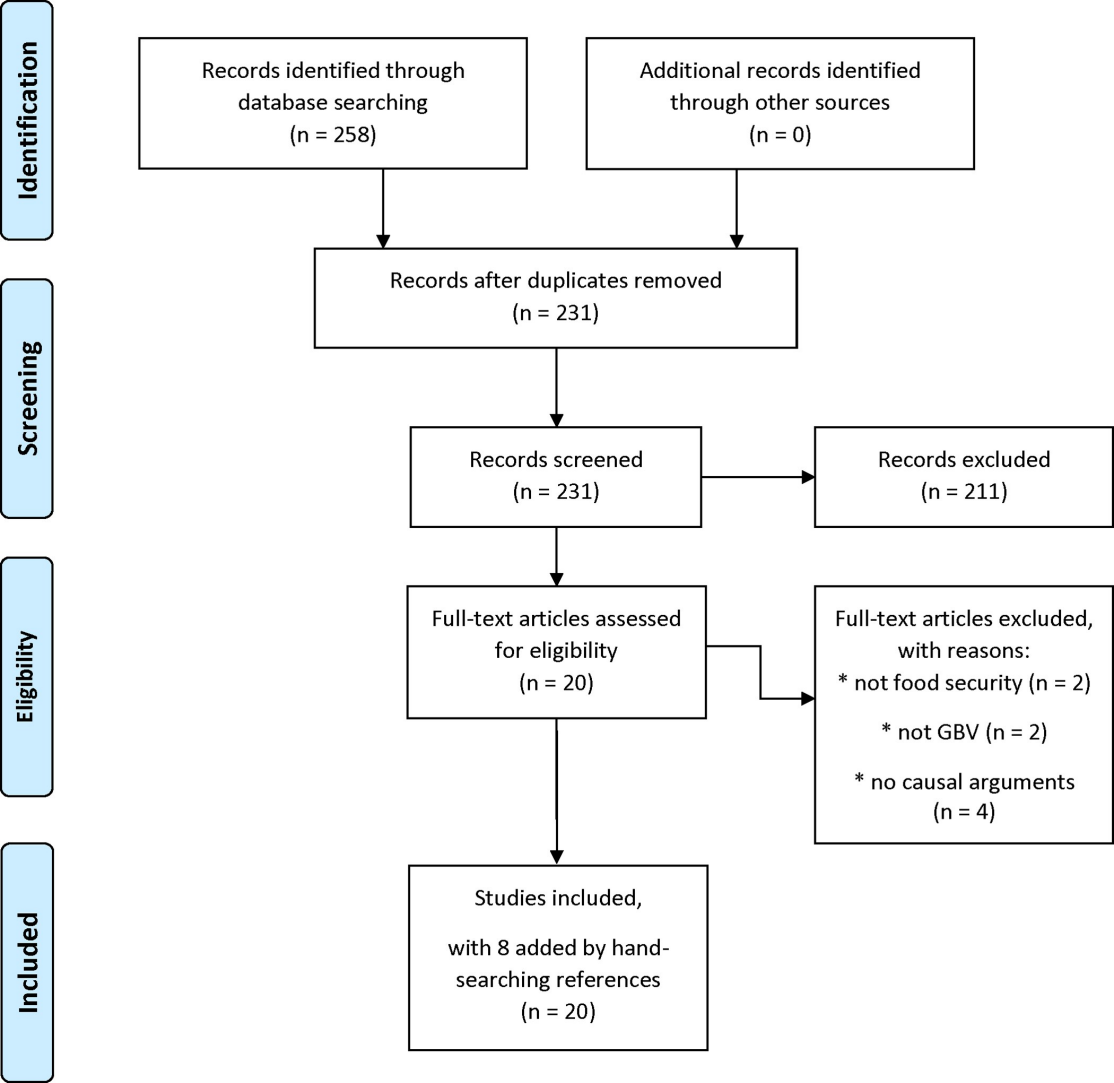
PRISMA flow diagram for scoping review investigating the interrelationship between food security, climate change, and gender-based violence (GBV).

Two authors (PA, BJB) reviewed the title, keywords, and abstract of all 231 de-duplicated records (Fig 1). Eight studies were additionally identified by manually examining the reference lists of all included articles. If no abstract was available and a decision could not be made otherwise, the authors searched online for additional information. The two authors then independently coded whether the record addressed the issue of *food security*. Next, these authors coded whether the record addressed the issue (or presumed effects) of *climate change*. The authors were also interested in concepts related to global warming. Finally, the authors coded whether the record addressed the issue (or presumed effects) of *gender-based violence*.

After independently coding each record, the two authors met to review coding decisions and resolve discrepancies. We computed the sum of the three coding decisions (1 = include, 0 = exclude). If the sum was equal to 3, then the record was deemed relevant and moved forward to full-text review. If the sum was equal to 2, *and* the gender-based violence concept was one of the two endorsed, then the record moved forward to the next phase of review. Otherwise, the record was excluded. Twenty full-text records were assessed for inclusion (Fig 1). Each included study for was assessed for risk of bias using the Newcastle-Ottawa Scale [39].

We used system dynamics modeling to accomplish our secondary objective, i.e., summarizing and disseminating scholarly findings to policy makers, funders, and researchers. System dynamics modeling, a valuable approach for transdisciplinary research [40,41], is the use of causal maps and computer simulation models to hypothesize, test, and refine endogenous explanations of ‘systems change’ [42], e.g., household food-security dynamics after a climatic shock such as severe flooding. System dynamics modeling is by its very nature a mixed methods approach and well suited to integrating diverse bodies of published evidence [40]. Thus, our final inclusion criterion was that the record had to include at least one text segment (e.g., sentence, contiguous sentences, paragraph) containing an argument asserting causal relationships between food security, climate change, *and* gender-based violence. After full-text records were assessed for inclusion and bibliographies hand-screened for additional references, we arrived at a final set of 20 records for extraction of results (Fig 1).

### Extraction of results

Extraction of results involved the creation of a “charting” table providing a descriptive summary of the results of the scoping review [31]. We used *Leximancer* content analysis software (Leximancer Pty. Ltd., Adelaide, Australia) to visually extract conceptual (presence and frequency of concepts) and relational meanings [43]. We created a concept map, representing the main concepts contained within the text as well as information about how they are related, to systematically obtain an overview of the material (S1 Fig).

Finally, we sought to integrate findings from this scoping review that covers a diverse body of published evidence related to global public health challenges and inequities associated with disaster events (e.g., climate shocks, pandemics, etc.), food insecurity, and GBV. This research reaches across scientific disciplines and regional boundaries. In order to integrate these diverse perspectives and advance knowledge, we adapted a published system dynamics model based on the COPEWELL (Composite of Post Event Well-Being) framework, intended to predict community functioning and resilience after disasters over time and identify opportunities for enhancing community resilience [44–46]. The conceptual definition of *community functioning* is “the ability and capacity of a community to provide a range of essential services to its inhabitants” [46].

The explicit and transparent nature of system dynamics modeling allowed us to integrate our scoping review findings and gain additional insights into the dynamics of and between disasters, food insecurity, and GBV. The spatial scale for the model is equivalent to a US county [46], of which there are currently 3,006 [47]. For a non-US example, the Republic of Kenya has 47 counties of various size [48], and research has documented how low-income households in rural Kenyan counties managed to maintain food expenditures post COVID-19 by reducing education expenditures [49]—which might be assumed, based on our scoping review findings, to disproportionately affect young girls (a form of domestic economic abuse). A key model assumption is that these geographic units (or communities) have the goal of maintaining their ability to provide a range of services to residents [46].

## Results

We document a relatively small but growing body of published evidence regarding the complex interrelationships between disasters (e.g. climate stressors), food insecurity, and gender-based violence (e.g. sex trafficking). Both food security and safety from gender-based violence can be conceptualized as subdomains of *community functioning*, and preliminary results from the conceptual system dynamics model illustrate the time course of community functioning in response to disasters.

Table 1 provides a charting of record details and characteristics from our scoping review of the interrelationship between food security, climate change, and gender-based violence. Given the paucity of published evidence regarding the interrelationship between these phenomena, publications from as early as 1982 were included. The focus of published literature varied widely—from the needs and struggles of urban poor during floods (i.e., climate shocks) [50] to women’s input into household decision making and their nutritional status in drought-prone countries (i.e., climate stressors) [51]. The empirical basis for most published literature was diverse (e.g., qualitative, quantitative, mixed methods). Eleven of 20 publications in Table 1 included empirical evidence but all were lacking in terms of strong causal study designs. The scales and levels of published literature were also diverse—from cities to slums (spatial scale), individual- to community- food security (social scale), and Latin America to South Asia (ecological scale). Almost every conceivable type of GBV has been associated with the relationship between climate shocks or stressors and food security—from domestic abuse (e.g., beatings, verbal abuse, economic coercion) to non-domestic abuse (e.g., harassment, trafficking, withholding entitlements). The specific forms of GBV in the final column of Table 1 (listed within parentheses) are for illustration purposes and not necessarily inclusive of all forms reported in the literature.

**Table 1:** Twenty articles identified for scoping review regarding the interrelationship between food security, climate change, and gender-based violence (GBV).

Author(s)	Focus^a^	Empirical Study?	Spatial Scale^b^	Social Scale	Ecological Scale	Temporal Scale	GBV Type(s)^c^
Rivers [52]	Essay on sex discrimination in disasters	No	Global	Individual, household food security	Global	Variable	* Domestic economic abuse (food allocation) * Domestic sexual abuse (early marriage)
Sapir [53]	Review of risks to female headed households and children without families in face of disasters	Yes -Secondary data analyses	Global	Individual, household and community food security	Global	Variable	* Non-domestic sexual abuse (sex for food)
Rashid [50]	Needs and struggles of urban poor during floods	Yes - Qualitative interviews (n = 32 men and women)	Cities (n = 1) Urban “slum areas” (n = 5)	Individual, household food security	South Asia (Dhaka City, Bangladesh)	Intra- and post-disaster periods (mid-July 1998 to September 1998)	* Non-domestic psychological abuse (harassment) * Domestic physical abuse (beating) * Domestic psychological abuse (verbal) * Domestic abuse (N/S)
Disasters Emergency Committee (DEC) [54]	Independent evaluation of effectiveness and efficiency of flood appeal	Yes -Qualitative interviews -Site visits	Countries (n = 1)	Individual, household, community food security	South Asia (Bangladesh)	Intra- and post-disaster periods (mid-July 1998 to September 1998)	* Non-domestic sexual abuse (N/S) * Domestic physical abuse (beating)
World Health Organization (WHO) [55]	Manual that defines how to develop reproductive health programs during each phase of conflict and displacement	No	Global	Individual, household and food security	Global	Intra- and post-disaster periods	* Non-domestic sexual abuse (sex for food) * GBV (N/S)
Haley [56]	Study of impact of drought and how it impacted mobile, highlands fringe people	Yes -Field work	Countries (n = 1) Zones (n = 1)	Individual, household and community food security	Australasia (Papua New Guinea)	Intra- and post-disaster periods (1997–1998)	* Domestic sexual abuse (early marriage) * GBV (witch killing)
Ariyabandu et al [57]	Handbook for mainstreaming gender dimensions of disaster risk management	No	Regions (n = 1)	Individual, households, community, national, and regional food security	South Asia	Variable	* Non-domestic economic abuse (underpaid) * Domestic economic abuse (inheritance laws) * Domestic sexual abuse (arranged marriages) * Non-domestic sexual abuse (relief camps) * Domestic physical abuse (wife battering)
Davis et al [58]	Special report of gender issues in tsunami recovery and planning	No	Regions (n = 1)	Individual, households, community, national, and regional food security	Southeastern Asia Southern Asia	Intra- and post-disaster periods	* Domestic abuse (N/S) * Non-domestic sexual abuse (rape, molestation, trafficking) * GBV (N/S)
Hindin [51]	Women’s input into household decisions and their nutritional status in three areas prone to drought	Yes -Quantitative surveys (n = 1,920, 2,870, and 6,219 women)	Countries (n = 3) Households (n = N/S)	Individual, household food security	Sub-Saharan Africa (Zimbabwe, Zambia, Malawi)	1999 Zimbabwe DHS; 2000 Malawi DHS; 2001–2002 Zambia DHS	* Domestic economic abuse (household decisions)
Buz [59]	Examined relationship between refugees and HIV/AIDS	No -Literature review	Global	Individual, household food security	Global	Variable	* Non-domestic sexual abuse (sex for food, rape) * GBV (N/S)
Dankelman et al [60]	Gendered analysis of how climate change impacts on human security	Yes -Literature review -Case studies -Secondary data	Global Countries (n = 3)	Individual, household, community food security	Global South Asia (Bangladesh) Sub-Saharan Africa (Ghana, Senegal)	Intra- and post-disaster periods (N/S)	* Domestic economic abuse (school withdrawal) * Domestic sexual abuse (early marriage / pregnancy, exploitation) * Domestic psychological abuse (verbal) * Domestic physical abuse (physical force) * Non-domestic psychological abuse (mental) * Non-domestic physical abuse (torture) * Non-domestic sexual abuse (harassment)
Food and Agriculture Organization (FAO) [61]	Cluster plan of action involving stakeholders involved in food security and agricultural livelihoods	Yes -Field consultation and analysis	Countries (n = 1) Zones (n = 1)	Individual, household, community food security	Sub-Saharan Africa (Northern Uganda)	Intra- and post-disaster periods (2008/09)	* Non-domestic sexual abuse (rape, exploitation) * Domestic economic abuse (inheritance, school withdrawal) * Domestic sexual abuse (early marriage) * Domestic physical abuse (battering) * Non-domestic economic abuse (employers)
World Health Organization (WHO) [62]	WHO summary of risks to health from climate change and what needs to be done	No	Global	Individual, household food security	Global	Variable	* Domestic abuse (N/S)
International Bank for Reconstruction and Development [63]	Analysis of social implications of climate change and climatic variability and options for improving resilience	Yes -Secondary analyses (municipal level data) -Field surveys	Regions (n = 2)	Individual, households, community, national, and regional food security	Latin America Caribbean	Variable	* Domestic abuse (N/S)
The Sphere Project [64]	Minimum standards to improve quality of humanitarian response in situations of disaster and conflict	N/A	Global	Individual, household, community food security	Global	Intra- and post-disasters period (N/S)	* GBV (N/S) * Non-domestic sexual abuse (trafficking, forced prostitution, rape, exploitation) * Domestic abuse (N/S) * Non-domestic economic abuse (entitlements)
Hasan [65]	Examines extent to which disasters affect social justice in relation to food security and nutrition	Yes -Secondary data (literature review) -Key informant interviews	Countries (n = 1)	Individual, household and community food security	South Asia (Bangladesh)	Variable	* Non-domestic sexual abuse (trafficking) * Domestic economic abuse (food allocation) * Non-domestic physical abuse (N/S) * Non-domestic psychological abuse (N/S)
Gurung et al [66]	Explores differences in impacts and adaptive capacities between and among women and men with respect to climate change	Yes -Literature review -Key informant interviews -In-depth interviews (n = 35) -Personal contacts (n = 76)	Countries (n = 1)	Individual, households, community, and national food security	South Asia (Nepal)	Variable	* GBV (N/S) * Non-domestic sexual abuse (rape, sex work) * Non-domestic psychological abuse (harassment) * Domestic economic abuse (fleecing)
World Health Organization (WHO) [67]	Review of the interactions between climate change, gender and health	No	Global	Individual, household food security	Global	Variable	* GBV (N/S) * Domestic abuse (N/S) * Non-domestic sexual abuse (harassment)
Vunisea et al [68]	Toolkit designed to support climate change practitioners in Pacific islands region to integrate gender dimension	N/A	Regions (n = 1)	Individual, household, community food security	Asia-Pacific	Intra- and post-disaster periods (N/S)	* GBV (N/S)
Food Rights Alliance [69]	Study establishing what is known about the effects of women’s exposure to shocks of vulnerability, poverty, and traumatic stressors of hunger and malnutrition	Yes (n = 312) -Literature review -Semi-structured, individual interviews -Focus group discussions -Household survey	Regions (n = 1) Districts (n = 2)	Individual, household food security	Teso Sub-Region (Eastern Uganda) Amuria and Ngora Districts	Variable	* Domestic sexual abuse (bride price) * Non-domestic economic abuse (land ownership) * Domestic economic abuse (household chores) * Domestic abuse (N/S)

^a^Scoping review inclusion criterion: The record had to include at least one text segment (e.g., sentence, contiguous sentences, paragraph) containing an argument asserting causal relationships between food security, climate change, *and* gender-based violence.

^b^N/S = non-specified.

^c^GBV types were assigned based on cross-classification of the nature of the victim-perpetrator relationship (domestic, non-domestic, non-specified) and the form of abuse (economic, physical, psychological, sexual, non-specified).

S1 Fig provides a ranked concept list (i.e., conceptual analysis [70]) of the top-ranking concepts from most to least frequent. “Concepts” are not simply words, but collections of related ideas that generally travel together throughout the text.

S2 Fig provides a concept cloud to plot the key concepts underpinning this area of scholarship as well as to clarify the conceptual boundaries of the topic (i.e., relational analysis [70]). Concepts are clustered into higher-level themes that are heat-mapped (e.g., red clusters ranked higher than orange clusters and so on). Perhaps not surprisingly given our scoping review protocol, the highest-ranking theme (red concepts cluster) was inclusive of concepts such as “women,” “food,” and “security.” The second highest-ranking theme (orange concepts cluster), which settled in the nearby map-space, was inclusive of concepts such as “disaster,” “resources,” and “risk.” The third highest-ranking theme (yellow-green concepts cluster) was inclusive of concepts such as “development,” “management,” and “adaptation”.

**Fig 2:**
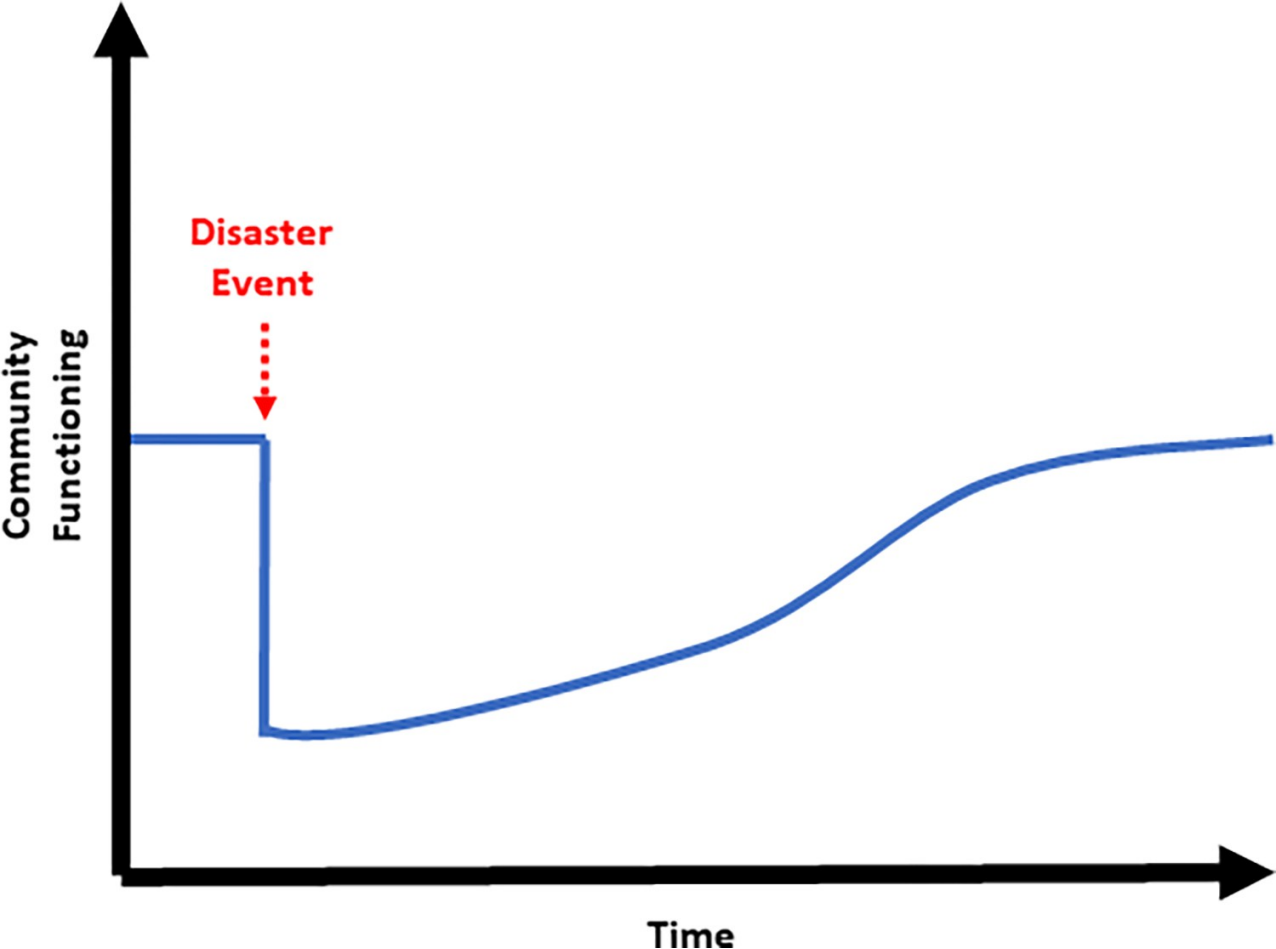
Reference mode of community functioning over time, before and after a disaster event (e.g., climate shock).

**Fig 3:**
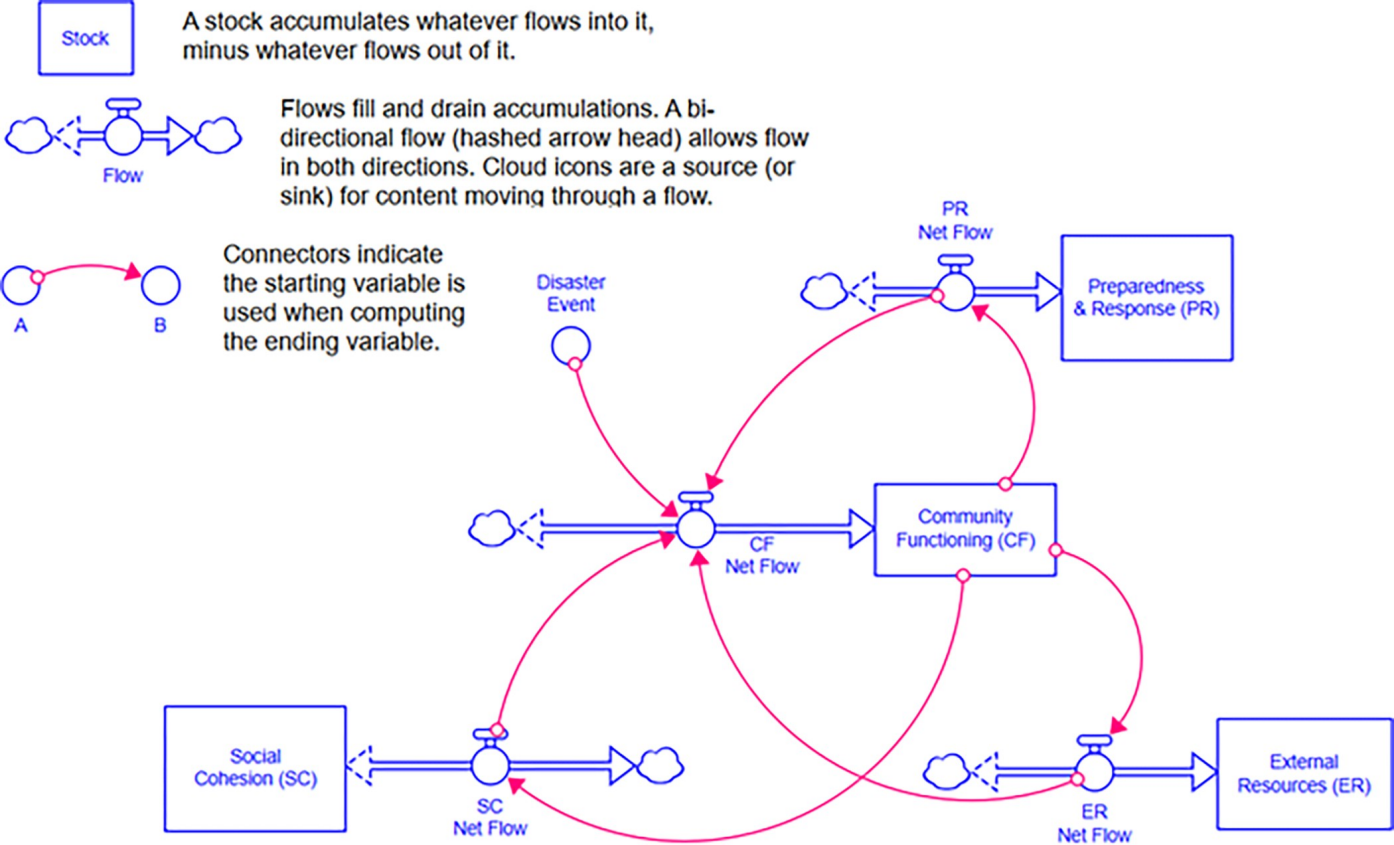
Conceptual system dynamics model of key components of community functioning (CF) after a disaster event.

Based on our system dynamics model, the main outcome of interest is community functioning, or the ability and capacity of a community to provide a range of essential services to its inhabitants, and how it varies over time [46]. Fig 2 displays the hypothesized model behavior to describe a community’s level of functioning, also referred to as a “reference mode”. Pre-event conditions are influenced by community functioning domains—e.g., community food and water, government (e.g., citizen safety), among others [46]. At some point in time, a disaster event occurs (e.g., climate shock, pandemic event) and community functioning plummets. Community functioning recovery after an event is determined by a set of system structures, multiple feedback loops in the system, and their reactions to and modification of the disaster event’s effects. It can be replenished over time from three sources: social cohesion; preparedness and response; and external resources. Resilience, a latent construct that cannot be measured ahead of a disaster event, is dynamic and comprised of two components: resistance and recovery [46]. A model demonstrating this dynamic hypothesis is shown in Fig 3.

**Fig 4:**
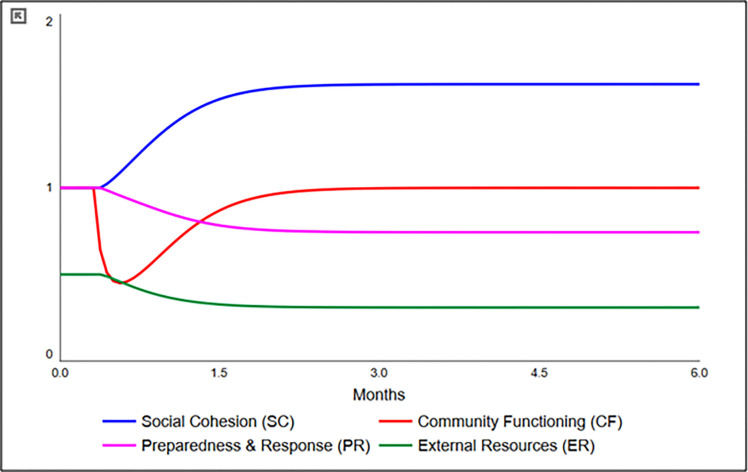
System dynamics model output, showing the depletion of the community functioning (CF) stock and replenishment from other stocks (SC, PR, ER).

The model output seen in Fig 4 demonstrates the time course of community functioning (CF) after an event, which then can be used to calculate resistance, recovery, and resilience for the geographic unit of interest [46]. In this conceptual model, a major disaster event at Week 1 reduces community functioning by approximately 50% very quickly but rebounds over time based on the contributions of preparedness and response (PR), social cohesion (SC), and external resources (ER). With further refinement, validation, and application, such a model “could improve decision-making by revealing ‘plausible futures’ after disasters” [46] and allow testing the sensitivity of the model to different policy solutions, such as those designed to prevent GBV in the face of increased food insecurity post-disaster events.

## Discussion

Based on the collection of texts reviewed here, the associations between climate shocks, food insecurity, and GBV have been explored, piecemeal, in many arenas over the past few decades. The complete picture of how each independently relates to the other and how all three intersect on a global scale, however, has not been fully described with empiric evidence. The COVID-19 pandemic, however, provides a real time and evolving global challenge that reinforces the interconnectedness of the three concepts presented here. The pandemic has illustrated how disasters can cause food insecurity, which can lead to more GBV. This scoping review aimed to identify and examine existing data on the topic, with a primary focus on the impact on gender-based violence.

We identified 20 scholarly references that discussed gender-based violence and either climate shocks or food insecurity. As expected, based on our search strategy, all articles included in this analysis described the interplay between all three concepts. The ranked concept list (S1 Fig) confirmed that appropriate articles were identified, as 75% or more of the context blocks were coded with “health”, “climate”, “women”, “food”, and “security”. The main concepts contained within the text, along with their associated themes, demonstrate the delineation of the conceptual boundaries of the topic (S2 Fig). This validates that the three main concepts identified above—climate change, food insecurity, and gender-based violence—are interconnected. We were able to address each of the stated objectives through this scoping review.

*Objective 1*: *Examine the extent*, *range*, *and nature of scholarly activity regarding the interrelationship between food security*, *climate change*, *and gender-based violence*. Several of the identified publications describe the interrelatedness of the three identified concepts in specific case studies. Qualitative studies of the impact of the 1998 floods in Dhaka City, Bangladesh [50,54] described how severe flooding (i.e. a climate shock) impacted basic infrastructure and livelihoods causing an increase in food prices and lack of safe drinking water (i.e. food insecurity). The resultant unemployment caused by the stressors of the flood led to increased tension within families and a rise in domestic violence. Haley [56] describes a 1997 drought that affected the Hawa people, an agrarian population in Papua New Guinea, leading to food insecurity and an increased incidence of “witch killings”, or violence predominantly targeted at women. A report by Dankelman et al [60] shows a similar phenomenon in response to disasters triggered by natural hazards in Senegal and Ghana, leading to sexual violence including rape, forced prostitution, early marriage of girls, and other forms of violence. These case studies are important contributions to the knowledge base and reveal not only the interconnectedness but causality between climate change, food insecurity, and GBV. Future research could strengthen our understanding by specifically incorporating causal methods such as process tracing and causal loop diagramming [71].

*Objective 2*: *Summarize and disseminate scholarly findings to policy makers*, *funders*, *and researchers*. While a data-driven definitive statement is premature, the judgment of these researchers is that climate shocks, a result of climate change, are associated with food insecurity. Of the twenty articles found to specifically address the interrelatedness between food security, climate change and GBV, the eleven empirical studies noted the same associations and trends. As climate change worsens, it leads to more variability and unpredictability in regional climates, resulting in more severe disruptions in the ability for populations to compensate. The stress of food insecurity may lead to higher levels of violence directed towards women, both within and outside of individual households. Psychological distress, financial dependence or fear of retribution towards themselves of their children may motivate women to stay with abusive partners [26]. Food insecurity can cause women and children to engage in unsafe or risky behaviors, increasing their risk for sexual violence and harassment while obtaining basic home goods such as food, water and firewood. All of these pressures are exacerbated by pandemics [20,26]. The timeliness of this potentially extensive crisis against women will hopefully compel policy makers, funders, and researchers to pay more attention to this interaction.

*Objective 3*: *Identify research gaps in the existing literature*. More targeted research is needed to generate direct evidence on the linkages between climate shocks (disasters), food insecurity, and the resultant impact on gender-based violence. The limited and heterogeneous literature we found discussing the interconnectedness of our identified concepts demonstrates that a full systematic review as well as a meta-analysis would have been premature. Each of the articles mentioned above highlights specific situations in a regional context and the direct impact on that particular affected population. In order to slow this trend or even reverse the effects at each phase of this three-pronged problem, and ultimately describe this in the global context, more attention, research, and resources are imperative. The topics and interactions examined here cut across politics, ethics, and science, which might partly explain a lack of studies of public health interventions that select climate action as their entry point. Primary research will likely allow for more-definitive conclusions based on evidence, to determine whether climate shocks leading to food insecurity and a subsequent increase in gender-based violence is a global phenomenon, as these authors postulate.

Our search strategy was comprehensive and involved a manual search through the reference lists of all included articles. However, given that our scope was global, there may have been articles that our search did not identify. The term gender-based violence typically refers to violence against women and girls, but few articles included men and boys as targets of attacks due to their gender and associated roles within a community. While we did not catalog the information retrieval strategies that were initially considered but rejected, a document that details our comprehensive search strategy is available from the corresponding author upon request. Additionally, one of our 20 sources was a hardcover book (Ariyabandu et al, 2005), which could not be uploaded into Leximancer for text analysis. Finally, given the recent impact of COVID-19, we replicated our initial search strategy within PubMed and located only five papers that would have been included had our initial search been performed now (July 24, 2021), none of which looked at the intersection of all three concepts or are in disagreement with our overall findings [72–76]. We created an analogous version of Table 1 that reflects a recent review of the interrelationship between pandemics, violence against women and children, and livelihoods (including food security) (Table 2) [26].

**Table 2:** Exemplar review article demonstrating the interrelationship between basic human needs (e.g., food security), pandemics (e.g., COVID-19), and gender-based violence (GBV) [26].

Focus	Empirical Study?	Relevant Segments	Spatial Scale	Social Scale	Ecological Scale	Temporal Scale	GBV Type(s)^a^
Economic Insecurity and Poverty-Related Stress	**No.** Used global literature, correlational evidence, causal estimates, analyzed studies.	“Even without direct shocks to earning levels, **pandemics** may incite temporary **food insecurity** and increased stress due to uncertainty about future **economic security** or general **well-being**. . .can lead to large increases in likely both the incidence and frequency of **IPV** and **violence** against children.”	“**Household** and **individual** level”	**Low-income** level. “location with weaker access to health and legal services” “economically depressed areas”	**Regional**. Specifically, an area that is poverty-stricken. “insecure populations tend to live in locations…” **Community**. Discussion of unemployment in men vs women.	Lifespan dependent on **economic security**	“Hypothesis hinges on expression of gender norms across settings”–**indicating both domestic and non-domestic violence**. Form of abuse is **economic.**
Quarantines and Social Isolation	**Yes.** Population-based telephone survey in Hong Kong.	“poor **mental health**, mental **disorders** and related factors, including alcohol **abuse**, have been shown to increase risk of **VAW/C**, with hypothesized effects both during and after times of **quarantine**”	**Household** level with mention of a “population-based study”	**Isolation** level (e.g. social isolation, functional isolation, physical/ geographical isolation)	**Community** level. Particular characteristic of isolation, mental-health issues, coping mechanisms, etc. in common.	Lifespan dependent on ability to **positively isolate**	Non-specified, but assumingly **domestic**. Form of abuse is mainly **psychological,** but risks of **physical**.
Disaster and Conflict-Related Unrest and Instability	**Yes**. Study exploring effects of the 2010 Haiti earthquake. Study of remote management strategies in northwest Syria.	“**pandemics** may result in the breakdown of societal **infrastructure**…may lead to increased exposure of women and children to **unsafe** and **risky settings**, including exposure to **sexual violence** and **harassment** during procurement of basic goods, including food, firewood, and water”.	**Population** level with mention of household level risks	**Displaced** (‘seeking asylum’ vs having asylum) level.	**Community** level. Characteristic of “relocation” and “family separation” in common.	Lifespan dependent on ability **secure asylum/shelter.**	**Non-specified.** Form of abuse include **sexual, economic, and psychological.**
Exposure to Exploitative Relationships due to Changing Demographics	**Yes.** Qualitative studies of orphans’ integration experiences in Lesotho and Malawi. Qualitative studies across sub-Saharan Africa.	“Increased **mortality, morbidity,** and fertility rates driven by **pandemics**….implications for the risks of **VAW/C** within extended family networks, as well as **exploitative relationships** for women and girls, especially those facing economic vulnerability.”	**Population** level. Specifically, young children and adolescent girls, such as orphans.	**Low-income** level (‘economic vulnerability’) **Relationship** (exploitative?) level.	**Community** level. Characteristic of being in an ‘exploitative relationship’ in common	Lifespan dependent on ability to leave **exploitative relationships**.	**Non-specified** (e.g. guardians of orphans may be related or not). Form of abuse is **economic, physical, psychological, and sexual.**
Reduced Health Service Availability and Access to First Responders	**No.** Systematic review of past pandemics and outbreaks	“The contraction of routine health services [due to **outbreaks** and **pandemics**] means **barriers** to screening and service provision for **VAW/C** will be amplified, including **reduced supply** of essential services for victims of **violence**, such as emergency contraception, post-exposure prophylaxis and psychosocial support”. “Evidence from the **COVID-19** pandemic suggests that women may **be less willing** to seek help, particularly for health care, because of perceived **risks** of contracting viruses”.	**Industry** level (health services)	**Essential Services** level.	**Community** level. Characteristic of wanting or needing services as a victim of violence in common.	Lifespan dependent on **available health services**	**Non-specified**. Form of abuse is non-specified but is assumingly **physical** or **sexual**.
Virus-Specific Sources of Violence	**Yes.** Mixed-method qualitative study of HIV positive individuals.	“Fears around the nature of specific **viruses** may create an **enabling** environment justifying the use of coercive and **controlling** behaviors, as well as more severe forms of **violence**”.	**Population** level (women and children)	**Control** level Displays of controlling behavior? Controlled to stay (ability to escape). **Low-income** level (‘economic insecurity’)	**Community** level. Characteristics including social stigmatization and being in controlling relationships in common.	Lifespan dependent on **social understanding of an ongoing virus.**	**Non-specified.** Form of abuse is **economic** and **sexual**. Mention of extreme cases that included forms of **psychological** and **physical** abuse
Inability to Temporarily Escape Abusive Partners	**Yes.** UNDP study assessing the response to gender-based violence during the Ebola epidemic.	“Women may opt to stay with **abusive** partners for a host of reasons, ranging from **emotional** attachment, **psychological** distress, financial **dependence**, or **fear** that separation will elevate **harm** to personal physical safety or the safety of their children…each of these may be compounded in **pandemic contexts**”.	“personal and familial constraints”. Overall **familial** and **household** level.	**Attachment** level Personal and familial constraints.	**Community** level. Characteristic of ‘ability to leave’ and complex relationships in common.	Lifespan dependent on **ability to escape abusive partners**	Non-specified, but assumingly **domestic**. Form of abuse is **economic**, **psychological**, and the immediate risk of **physical.**
Exposure to Violence and Exploitation in Response Efforts	**Yes.** Population-based study in parts of South Sudan. Participatory action, longitudinal qualitative study of SEA in relation to aid distributions in Lebanon and Uganda. Aid assessment from Sierra Leone.	“increased pressure on WASH **resources** in this **pandemic** may also lead to increased **exploitation** by responders, as well as other types of **VAW/C**, with female- or child-headed households particularly at **risk**”.	**Population** level (e.g. against refugees)	**‘Need for’ or ‘access to’ aid** level. (e.g. food, WASH, shelter, cash, voucher assistance, fuel, firewood)	**Community** level. Characteristic of needing items (such as food shelter, etc.) to live.	Lifespan dependent on **response efforts to decrease violence exposure.**	**Non-domestic**. Form of abuse is **physical** and **sexual** fueled by **economic** hardship.
Violence Perpetrated against Health Care Workers	**Yes.** Verbal abuse sample from University Hospitals in South Korea and Germany.	‘In **emergency** settings, female health workers are at **heightened risk** to both routine and severe **violence**’.	**Population** level (female healthcare workers)	**Gender (equality and stigmatization)** level.	**Individual** level, group/class of female health care workers.	Lifespan dependent on **stigmatization of female health care workers.**	**Non-domestic.** Forms of abuse are **verbal** and **physical**.
Other Potential Pathways	**Yes**. Media reports in Seattle. Nationally representative data from the United States.	‘other forms of **generalized violence** are important, and may occur during **pandemics**, including elder **neglect** and **abuse**, child online **solicitation** and abuse, or **hate crimes** driven by **xenophobia** (all potentially relevant to COVID-19)’.	**Population** level	**‘Ability to bear firearms’** level (for first half) And **generalized violence** level	**Community** level. Characteristic of facing generalized violence in common.	Lifespan dependent on **violence norms and xenophobia**	**Non-specific**, mention of both **domestic** and **non-domestic** violence. Forms of abuse are **emotional** and **physical**

^a^GBV types were assigned based on cross-classification of the nature of the victim-perpetrator relationship (domestic, non-domestic, non-specified) and the form of abuse (economic, physical, psychological, sexual, non-specified).

## Conclusion

A small but growing body of global public health literature reveals simple and dyadic connections between climate shocks, food insecurity, and gender-based violence. The interconnectedness of all three concepts, however, begins to paint a more complete picture. Better insight into the causal mechanisms and related concepts, such as community functioning, resilience, and recovery can further our understanding into these nuanced interactions. More causal research and focused interventions can help curb the tide of the potentially devastating and synergistic phenomenon of climate shocks leading to food insecurity and a subsequent increase in gender-based violence. As researchers achieve a deeper understanding of the multifaceted impact of COVID-19, a global stressor that interacts with each of the three concepts, it will likely prove demonstrative in its permeation through all aspects of global vulnerabilities.

## Supporting information

S1 Fig Ranked concept list regarding the interrelationship between food security, climate change, and gender-based violence. (TIFF)

S2 Fig Concept cloud to map the interrelationship between food security, climate change, and gender-based violence. (TIF)

S1 File Database search strategies investigating the interrelationship between food security, climate change, and gender-based violence. (DOCX)
