# Supplementary figures and images for: The interrelationship between food security, climate change, and gender-based violence: A scoping review with system dynamics modeling
Source: PLOS Glob Public Health. 2023 Feb 24;3(2):e0000300. doi: 10.1371/journal.pgph.0000300 (PMC10021784; doi:10.1371/journal.pgph.0000300)

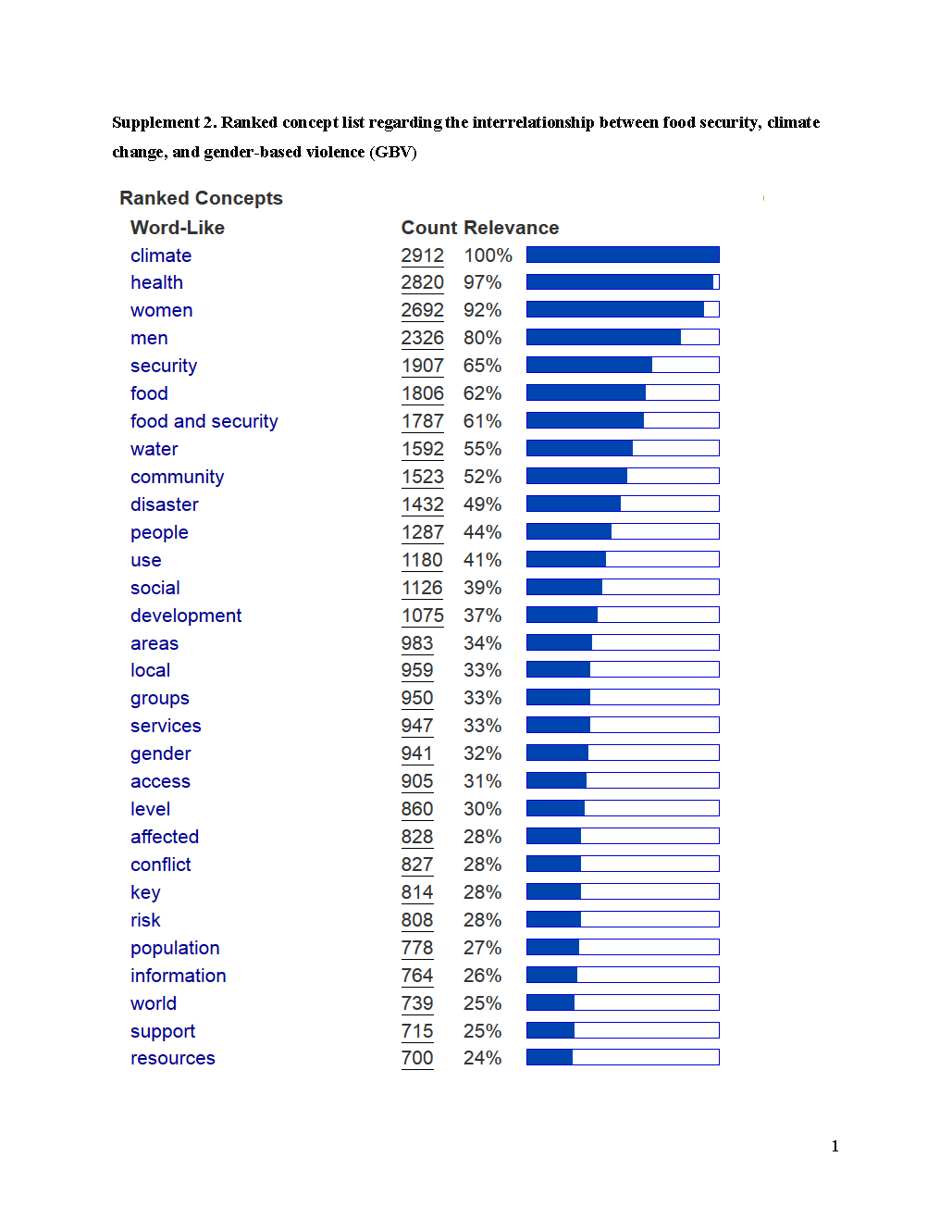

Supplement: S1 Fig — (TIFF) [file pgph.0000300.s001.tiff]

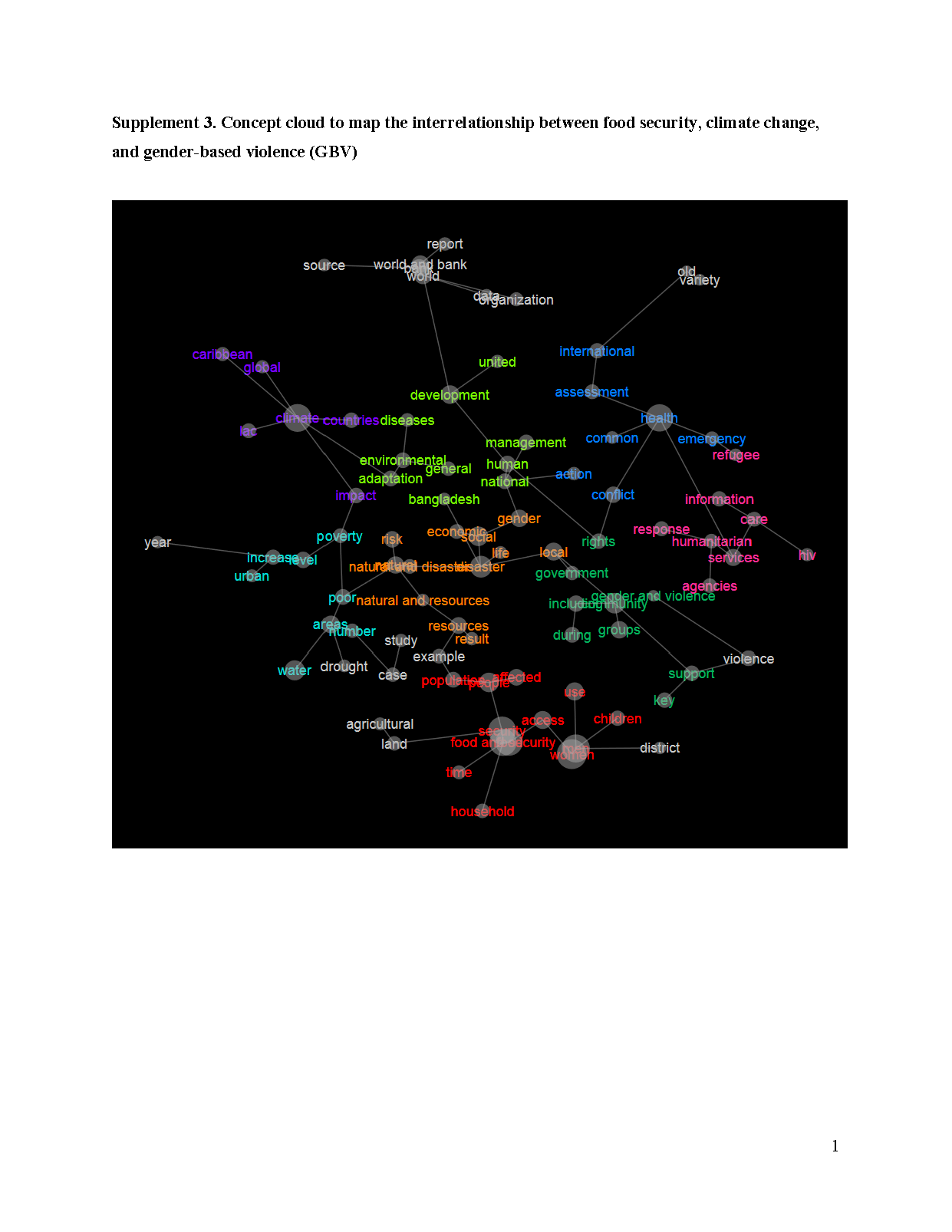

Supplement: S2 Fig — (TIF) [file pgph.0000300.s002.tif]
