# Supplementary material for: The interrelationship between food security, climate change, and gender-based violence: A scoping review with system dynamics modeling
Source: PLOS Glob Public Health. 2023 Feb 24;3(2):e0000300. doi: 10.1371/journal.pgph.0000300 (PMC10021784; doi:10.1371/journal.pgph.0000300)
Supplement: S1 File — (DOCX) [file pgph.0000300.s003.docx]

**Supplement 1. Complete search terms, formatting adapted for each database**

domestic violence OR child abuse OR spouse abuse OR rape OR sexual abuse OR sexual assault OR sexual harassment OR violence OR social inequalities OR aggressive behavior OR aggressive behavior

AND

food insecurity OR water insecurity OR water security OR water insecurity OR food supply OR food supplies OR water supply or water supplies OR food scarcity OR water scarcity OR food accessibility OR food inaccessibility OR water accessibility OR water inaccessibility OR food availability OR food unavailability OR water availability OR water unavailability OR starvation OR famine OR water supply

AND

climate change OR drought OR landslides OR earthquakes OR floods OR fire damage OR fire OR climate shock OR climate swing OR extreme weather OR severe weather OR extreme heat OR heat wave OR severe heat
